# Inkjet-Assisted Electroformation of Magnetically Guidable Water Striders for Interfacial Microfluidic Manipulation

**DOI:** 10.1021/acsami.2c17792

**Published:** 2022-12-13

**Authors:** Roberto Bernasconi, Davide Carniani, Min-Soo Kim, Salvador Pané, Luca Magagnin

**Affiliations:** † Dipartimento di Chimica, Materiali e Ingegneria Chimica “Giulio Natta”, Politecnico di Milano, via Mancinelli 7, 20131Milano, Italy; ‡ Multi-Scale Robotics Lab, Institute of Robotics and Intelligent Systems, ETH Zurich, Tannenstrasse 3, CH-8092Zürich, Switzerland

**Keywords:** microrobots, water striders, surface tension, electroforming, inkjet, remote control

## Abstract

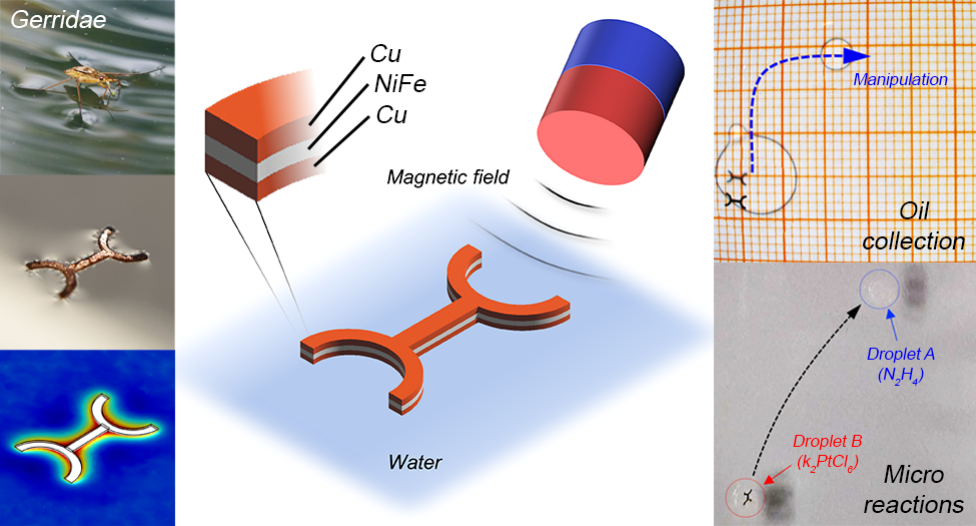

*Gerridae*, colloquially called water striders, are a peculiar class of insects characterized by the extraordinary ability to walk on the surface of water bodies. Owing to this capacity, they constitute an ideal source of inspiration for designing untethered microdevices capable of navigating the interface between two fluids. Such steerable micrometric objects can be of great interest for various applications, ranging from the handling of floating objects to the remote control of microreactions and the manipulation of self-assembled monolayers. This paper describes the realization of artificial water striders via an inkjet-assisted electroforming approach. Inkjet deposition patterns the negative mask, which is subsequently filled with different layers of metals through electroforming. One of such layers is the magnetic alloy NiFe, which allows wireless propulsion of the striders by means of externally applied magnetic fields. The magnetic actuation tests prove good maneuverability at the water–air and silicone oil–air interfaces, with superior control over the speed and position of the devices. The surface of the devices is modified to tune its superficial energy in order to maximize buoyancy on these different combinations of fluids. A magnetic field-controlled strider manipulates a droplet and demonstrates collecting oil microdroplets and synthesizing platinum nanoparticles by chemical microreactions. Finally, the remotely operated microrobot could be employed in laboratories as a real avatar of chemists.

## 1 Introduction

Nature has traditionally been a major source of inspiration for the development of miniaturized devices able to operate at the microscale to carry out specific tasks.^1,2^ Indeed, creatures living in the microworld developed, during billions of years of evolution, peculiar strategies to live and move at a scale in which surface forces are predominant. Constrained by the necessity of developing nonreciprocating actuation strategies, in accordance with the scallop’s theorem enunciated by Purcell,^3^ researchers face analogous challenges in the development of artificial untethered microrobots.^4^ Consequently, it is a natural choice for them to take inspiration from bacteria, protozoans, or small insects for their realization. Following this approach, bioinspired functional microdevices for a wealth of different applications, both in vivo and ex vivo, have been developed in the last few years. Literature examples include microrobots for active cells^5−7^ and drug delivery,^8,9^ for remote micromanipulation,^10^ cancer therapy,^11^ diagnosis,^12^ or even environmental remediation.^13^ Two strategies have been proposed to move microrobots at the microscale and carry out these functionalities: the use of chemical propulsion^14^ or the exploitation of an external source of energy (light,^15^ ultrasound,^16^ or magnetic fields^17^). The first methodology relies on the presence of a suitable chemical fuel dissolved in the environment in which the microrobot moves. However, such fuel is normally not naturally present in the environment, and its introduction poses serious challenges in terms of biocompatibility (in the case of in vivo applications^18^) or environmental contamination (in the case of environmental applications). Regarding the use of external power sources, the most promising methodology is probably the use of controlled magnetic fields. By applying magnetic materials on the devices, it is possible to wirelessly control microdevices with great precision and with virtually no interactions with living tissues and nonmagnetic materials. According to the geometry of the device and to the nature of the materials employed, different magnetic patterns can be applied to actuate the microdevices: gradients,^19^ oscillating field,^20^ and rotating fields.^7,13^

One of the most interesting environments in which a magnetically guided microrobot could move is the interface between two fluids. Basically, the fluid–fluid interface can be assimilated to a bidimensional environment, in which the two fluids manifest properties that are often different from their bulk phases.^21^ At the interfaces, surface energy and diffusion/evaporation play a major role in the chemical phenomenon. In addition, some molecules and nanoparticles (NPs) show the ability to self-organize as ordered patterns (allowing techniques like, for example, self-assembled structure formation^22^ or nanosphere lithography^23^). Furthermore, the properties of fluid–fluid interfaces can be exploited for advanced chemical reactions.^24,25^ Finally, interfaces can be simply used as supports for the controlled motion of droplets of a third phase.^26,27^ Considering the importance of fluid–fluid interfaces, it is desirable to develop microdevices able to manipulate them. In this context, the most obvious source of inspiration for a magnetically moveable microdevice able to run on the surface of a fluid is represented by the insects of the genre *Gerridae*. All the members of this family are characterized by a singular ability: they walk on the surface of the water–air interface exploiting surface tension.^28^ For this reason, they are commonly called water-striders. In fact, Gerridae present long legs, which distribute the weight of the insect on a wide surface and exploit the buoyancy effect connected to surface tension to counterbalance gravity. In a similar fashion, an artificial water-strider should be characterized by a flat shape able to distribute the weight on a wide contact area with the fluid. Contrarily to real water-striders, its motion can be based on the controlled application of external magnetic fields. A magnetically guided water-strider can be potentially used to carry out a wealth of different tasks. It can be employed, for example, to transport loads^29,30^ and manipulate floating objects.^31−34^ It can collect or manipulate droplets of fluids at the interface between two other fluids, harvest energy from the surface of water,^35^ and carry out environmental monitoring.^36^ In addition, a floating device can potentially manipulate self-assembled layers of molecules or NPs during their formation.^37^ The controlled manipulation of small fluid volumes, in particular, is of interest for a wide variety of applications like biological sample handling^38,39^ or chemical reactions.^40^ As a proof of the attractiveness of these applications, a wealth of droplet manipulation methodologies has been developed. These are based on acoustofluidic manipulation,^41^ mechanical handling,^42^ programmed wettability,^43^ electrowetting displacement,^44^ and untethered actuation of microrobots.^45^

In this paper, we aim to realize bioinspired artificial water striders, able to run on the surface of a fluid propelled by a magnetic field and to carry out micromanipulation tasks. For their production, a smart combination of inkjet printing and electroforming is exploited for the first time. Inkjet printing is a highly attractive, up to date almost unexplored, technique for the realization of untethered microdevices. Through the controlled deposition of droplets, it presents interesting patterning capabilities at the microscale that can be exploited to build functional structures. It is costless and fast and allows optimal material usage. In addition, material jetting can also be used to perform reactive inkjet printing, a technique that allows the in situ formation of a compound starting from two materials jetted from two distinct solutions. This capacity considerably widens the selection of usable materials, and the few examples of inkjet manufactured untethered microdevices available in the literature have been produced using this approach.^46,47^ Nevertheless, inkjet also presents some drawbacks, especially when applied to the patterning of the materials required for magnetic actuation. The deposition of magnetic materials can be carried out by jetting NP suspensions,^48^ by printing polymer-NP composites,^49^ or by printing sol–gel precursors.^50^ All these methodologies present specific problems (cost, quality of the result, etc.), and it is, in most cases, difficult to obtain magnetic properties similar to bulk metals or alloys. Taking this into consideration, the present work implements a printing-electroforming approach, where a negative polymeric mask is patterned on a conductive substrate and then metal is subsequently electroformed in the mask to obtain the positive shape of the devices. By following this approach, artificial water striders characterized by high magnetic properties can be easily obtained. Their morphological features and their motion at the water–air interface are investigated. To demonstrate their potential, two applications are hereby developed: oil collection on the surface of water^51^ and a microreaction for the synthesis of NPs.

## 2 Experimental Methods

### 2.1 Materials

SU-8 2005 was acquired from Microchem. AF1600 was purchased from DuPont and suitably diluted with the solvent FC40 (obtained from 3 M) following the guidelines of the manufacturer. All the remaining chemicals employed during the experimentation were acquired from Sigma-Aldrich and used as received.

### 2.2 Substrate Preparation

Small plates of Al 2011 alloy were employed as substrates. Their dimensions were as follows: 50 mm as length, 25 mm as width, and 1.5 mm as thickness. To get a uniform surface, the surface of the substrates was polished with sandpaper (number 100, 600, and finally 1200). Then, the surface was cleaned in acetone under sonication and water to remove the residues resulting from the polishing process. After a drying step with nitrogen, the plates were immersed in HNO_3_ 50 wt % to remove surface oxide. The surface was then dried with nitrogen and corona-treated for 5 min.

### 2.3 Polymeric Mask Inkjet Printing

To pattern the polymeric negative mask, a single layer of SU-8 was inkjet printed on the Al substrate following an approach available in the literature.^52^ Briefly, SU-8 2005 was mixed at 40 wt % with cyclopentanone, and the resulting solution was loaded in a Dimatix DMP printer. 20 V was selected as the actuation voltage for the nozzles, drop spacing was set to 20 μm, plate temperature was set to 60 °C, and cartridge temperature to 40 °C. At the end of the printing process, the SU-8 layer was annealed placing the Al plate on a hot plate and following the standard procedure indicated by the manufacturer: 1 min of prebake at 65 °C, 2 min of soft bake at 95 °C, exposure to UV light for 2 min, and postexposure bake for 1 min at 65 °C and then for 1 min at 95 °C. All these steps, except UV exposure, were carried out in the dark. UV light was generated from a lamp containing four fluorescent UV bulbs (9 W nominal power).

### 2.4 Electroforming Procedure

To electrodeposit on the Al alloy inside the SU-8 pattern, the substrates were subjected to the zincate process. The following solution was employed: NaOH 380 g L^–1^, ZnO 75 g L^–1^, KNaC_4_H_4_O_6_·4H_2_O 10 g L^–1^, FeCl_3_ 1 g L^–1^, CuSO_4_·5H_2_O 5 g L^–1^, and NaC_12_H_25_SO_4_ 1 g L^–1^. The Al plate coated with the SU-8 pattern was immersed for 1 min in the zincate solution, extracted and washed, immersed in HNO_3_ 50 wt % for 1 min, extracted and washed, immersed for 1 min in the zincate solution, extracted and washed. The first copper layer was deposited from a pyrophosphate electrolyte having the following composition: CuSO_4_·5H_2_O 120 g L^–1^, K_4_P_2_O_7_ 240 g L^–1^, and KNO_3_ 5 g L^–1^. The pH of the bath was corrected to 9 with NH_4_OH, and the deposition was carried out at 50 °C and 10 mA cm^–2^ for 50 min (under stirring). The NiFe layer was deposited from the following electrolyte: FeSO_4_·7H_2_O 14 g L^–1^, NiSO_4_·7H_2_O 200 g L^–1^, Na_3_C_6_H_5_O_7_ 20 g L^–1^, NaCl 25 g L^–1^, H_3_BO_3_ 40 g L^–1^, NaC_7_H_4_NO_3_S 3 g L^–1^, and NaC_12_H_25_SO_4_ 0.3 g L^–1^. The pH was set to 3.5 using sulfuric acid, and the deposition was carried out at 55 °C and 10 mA cm^–2^ for 45 min (under stirring). The second copper layer was deposited at room temperature, at 10 mA cm^–2^ and for 45 min using the commercial solution Cuproplus (by Tecnochimica). The bath was stirred.

### 2.5 Postelectroforming Steps

At the end of the electroforming process, SU-8 was removed from the surface of the Al plates by a 1-day immersion in *N*-methyl-2-pyrrolidone. Finally, the devices were released from the substrate by immersing them in a 2 M solution of NaOH. After a few hours, released devices were collected from the solution, washed with water, and dried with nitrogen. To control the wettability of their surface, some devices were either coated with a fluoropolymer or oxidized in air. In the first case, a 0.6 wt % solution of AF1600 fluoropolymer in FC40 solvent was employed. The devices were placed on a grid, immersed in the solution, extracted, dried in air, and finally annealed at 120 °C. In the case of controlled oxidation, the devices were heated at 200 °C for 10 min in air.

### 2.6 Sample Characterization

Optical microscopy (OM) was carried out using a Leica DM LM microscope. Scanning electron microscopy (SEM) and energy-dispersive spectroscopy (EDS) were performed using an EVO 50 EP scanning electron microscope (by Zeiss) and an Inca Energy 200 EDS module (from Oxford Instruments). X-ray diffraction (XRD) was performed by means of an Xpert MPD setup (by Philips, in thin film mode and with Cu *K*_α_ = 1.5406 Å). Magnetic characterization was carried out using a MicroMag 3900 (from Princeton Measurement Corp.) vibrating sample magnetometer (VSM). XRF measurements were performed by means of an X-RAY XAN apparatus (by Fischerscope). The instrument used to get atomic force microscopy (AFM) topographical data was a Solver Pro (by NT-MDT). A CM 10 setup was employed to acquire transmission electron microscopy (TEM) images.

### 2.7 Magnetic Actuation

To characterize actuation performance, a single device was placed inside a glass basin having an internal diameter of 57 mm and a height equal to 8 mm. Such basin was filled with water for half its volume, which corresponded to 10.2 mL of fluid. The basin was placed in the middle of an array of eight electromagnets, called OctoMag,^53^ able to apply uniform, rotating, and oscillating magnetic fields as well as gradients along five DOFs. Gradients between 1 and 30 mT m^–1^ were applied to move the devices, while constant fields between 1 and 5 mT were applied to control their direction. The videos of the actuation tests were acquired with an external camera. To determine the speed of the devices, position vs time data were acquired from these videos employing the software Tracker (by OSP).

### 2.8 Oil Collection Tests

To carry out oil collection tests, the same basin previously described was employed. It was placed in the OctoMag, and small droplets of silicone oil (viscosity 20 cSt at 25 °C; density 0.95 g mL^–1^ at 25 °C) were dispensed on the surface of the water and collected using a single device.

### 2.9 Microreaction Experiment

To perform a prototypical microreaction experiment, the synthesis of Pt NPs, the basin was filled with AP100 silicone oil (viscosity 100 mPa s at 25 °C; density 1.065 g mL^–1^ at 20 °C). A 50 vol % ethanol solution was mixed and used to prepare two solutions, namely, A and B. Solution A contained 1 mM K_2_PtCl_6_, while B contained 8 mM N_2_H_4_·H_2_O. Then, 50 μL of solution A were dispensed on the surface of the AP100 silicone oil to form a floating droplet. Twenty-five microliters of solution B were dispensed in a separate floating droplet. A single device was placed inside the droplet of solution A and magnetically steered toward the droplet of solution B. When the two droplets merged, the resulting droplet was collected with a micropipette. To establish a comparison, the same reaction was carried out at the macroscale mixing 10 mL of solution A with 5 mL of solution B in the absence of stirring.

## 3 Results and Discussion

### 3.1 Artificial Water Strider Design and Actuation Principle

**Figure 1:**
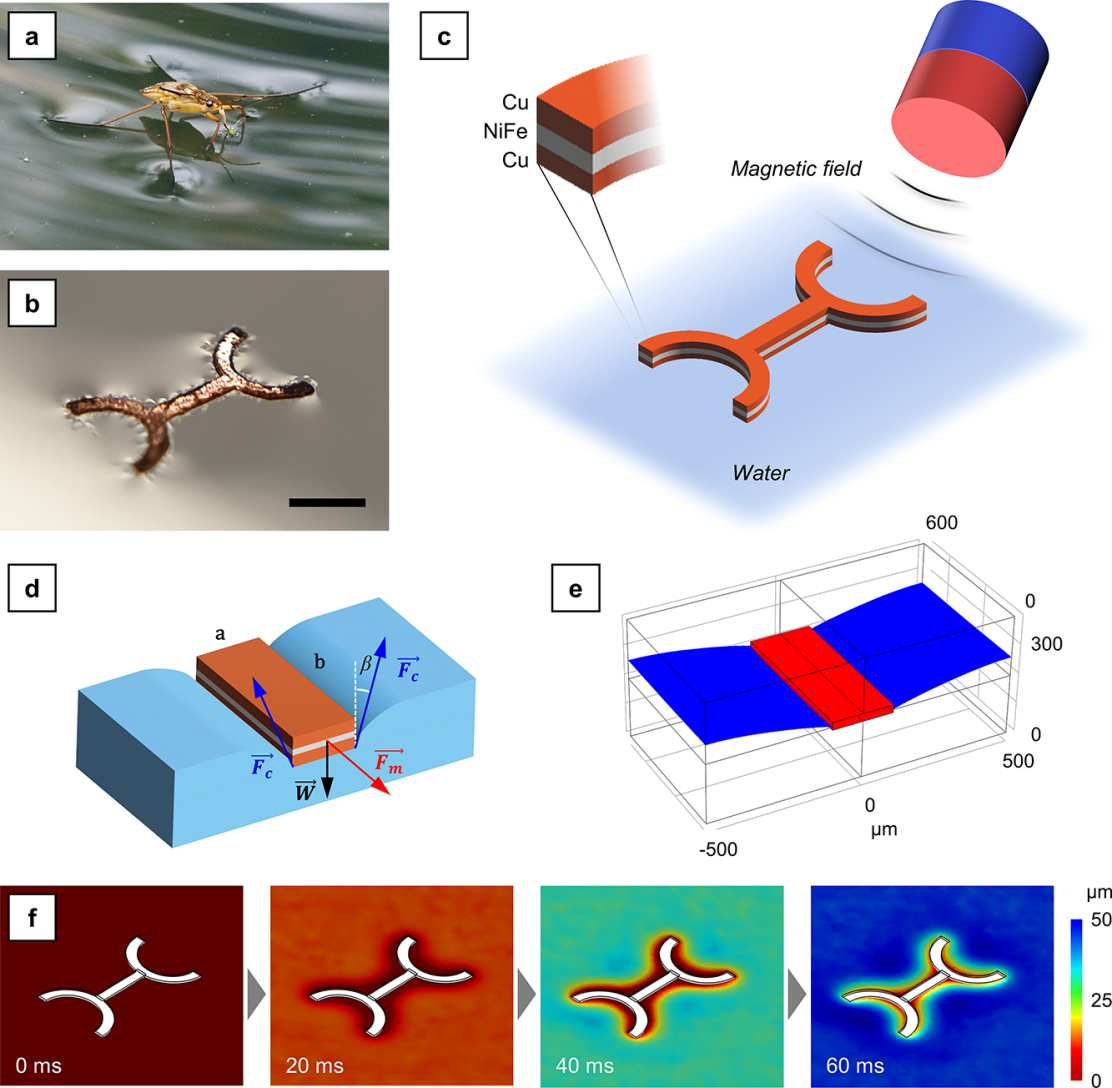
Schematic diagram and design of the bioinspired artificial water strider. (a) Insect of the *Gerridae* family. (b) Developed artificial water strider (scale bar: 1 mm). (c) Schematic diagram showing magnetic field control of the artificial water strider. (d) Force body diagram on a single device. (e, f) Finite element method (FEM) simulation results of the wetting process of an artificial water strider.

To properly design an artificial water strider inspired by the insects of the genre *Gerridae*, it is fundamental to understand how these creatures exploit surface tension to float on the surface of water ponds and how they propel. *Gerridae* are a family of insects of the order *Hemiptera* that are classified as pleuston, a term that indicates the air/water interface of a body of water as their natural habitat (Figure 1a). *Gerridae* do not exploit Archimedes’ principle to float on water. In place, they exploit the relatively high surface tension of water.^28^ Water striders present highly adapted body features in the form of long, hydrophobic legs. By using these body parts, they are able to distribute their weight on a great surface and to increase the contact angle (by lowering down the surface energy) of the surface that goes in contact with water.^54,55^ The force generated by surface tension counterbalances the weight of the insect, generating a visible deformation of the water/air interface under the legs of the water strider (Figure 1a).

Inspired from water striders, which can walk at water–air interfaces, the artificial water strider was developed to navigate at the two-fluid interface (Figure 1b). Figure 1c shows a schematic diagram of an artificial water strider, which is a flat device having a central body and four legs (detailed design parameters in Figure S1 and Table S1). The central linear part mimics the body of the real strider, while the semicircular parts attached to the central body mimic the elongated middle and back legs of the insect. Such legs increase the extent of the zone in contact with the water and provide stability to the device, allowing it to float on the water in a stable way (without rolling). The artificial water strider has three layers of Cu/NiFe/Cu, and the NiFe magnetic layer enables its actuation by external magnetic field. To verify its floating mechanism based on the design, forces acting on the device were evaluated.^56−58^ First, capillary force^56^ can be quantified using eq 1.
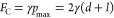
1

The capillary force *F*_C_ exerted on the cylinder is proportional to the surface energy between the fluid and the surface of the cylinder (γ) and to the external maximum perimeter *p*_max_ of the cylinder. The latter corresponds to two times the sum of the diameter *d* and the length *l* of the cylinder itself. The devices hereby presented, however, have been produced via electroforming on a patterned surface. Consequently, they presented a flat morphology that makes them more similar to shaped parallelepipeds that to cylinders. In the case of a parallelepiped floating on a fluid (Figure 1d), the expression for capillary force is similar^56^ (eq 2).
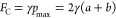
2

In this case, the perimeter of the object corresponds to twice the sum of the two dimensions (*a* and *b*) of the parallelepiped parallel to the air/fluid interface. Obviously, the vertical component of the capillary force counterbalances the weight *W* (mg; mass multiplied x gravitational acceleration), resulting in the equilibrium condition described by eq 3.
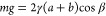
3

By looking at eq 3, it is evident that the perimeter of the devices should be as high as possible with respect to the weight of the devices, justifying thus the choice of creating the legs on the device.

From the dimensional point of view, the dimensions of the devices were selected to allow efficient printing and proper manipulation at the air/water interface. Finally, the external perimeter is equal to 10.57 mm. This value allows roughly evaluating the maximum weight of the device that can be sustained by the water. A priori, the angle β is unknown and corresponds to the value required to exactly counterbalance the weight *W*. However, the maximum value for the surface tension contribution is achieved when the cosine of β is 1 and β itself is 0°. Under this condition, assuming γ equal to that of pure water at 25 °C (71.97 mN m^–1^), the maximum weight of the device can be equal to 77.53 μg. Beyond this value, β would become negative and the water would submerge the device.

Taking into account this weight limitation, the thickness and the composition of the metallic layers that constituted the devices were determined. For propulsion, *Gerridae* move on the surface of water ponds by exploiting the rowing action of their legs. In the case of their artificial counterparts, propulsion can be provided by applying a gradient of magnetic field. The magnetic force *F*_m_ applied^2^ is expressed by eq 4.
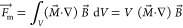
4*M* is the magnetization, *B* is the field applied, and *V* is the volume of the magnetizable material. To apply a magnetic force, the devices must contain a ferromagnetic material. For this reason, NiFe was used as a functional material to electroform the devices. Electrodeposited NiFe is, in most cases, brittle and highly stressed. For this reason, the NiFe layer was enclosed between two copper layers that acted as structural supports. Furthermore, Cu can be easily deposited with excellent surface finish from additivated commercial electrolytes (like the Cuproplus bath employed in the present work), yielding thus a smooth finish to the external surface of the device.

Since the force applicable to the devices depends on the volume of the magnetic material (eq 4), the volume of NiFe was kept relatively high with respect to the volume of Cu. The following sequence of layers was designed: 10 μm Cu, 6 μm NiFe, and 10 μm Cu. By doing this, the final nominal volume of magnetic material (NiFe) was 30% of the volume of the nonmagnetic material (Cu) and 23.1% of the global volume of the device. The final expected weight of the device was 23 μg, which is well below the limit previously determined.

The behavior of a single device placed in contact with water was evaluated carrying out a simulation by means of the FEM. Figure 1e depicts the result obtained from an analogous simulation, which was performed considering only a section of the device. The final level reached by the water was roughly 70 μm above the bottom surface of the device. Indeed, the device was put in contact with water, and the level of the water was progressively raised (Figure 1f). The steady condition was reached when the pressure exerted by the fluid counterbalanced the weight of the device (considered equal to 23 μg). The contact angle between the device and water was set equal to 2 rad (114.59°). Figure 1f depicts the result obtained with respect to time. The water level, measured with respect to the bottom face of the device, progressively raised and reached the steady condition after roughly 60 ms.

### 3.2 Device Production

The production of the devices was based on a combination of patterning and electroforming steps. Aluminum was employed as a substrate because it can easily be etched in an NaOH solution to detach the devices. Opportunely, all the other metals used during the process (Cu, Ni, and Fe) passivate in alkaline conditions. Thanks to this, it was possible to selectively dissolve Al and detach the devices from the substrate.

**Figure 2:**
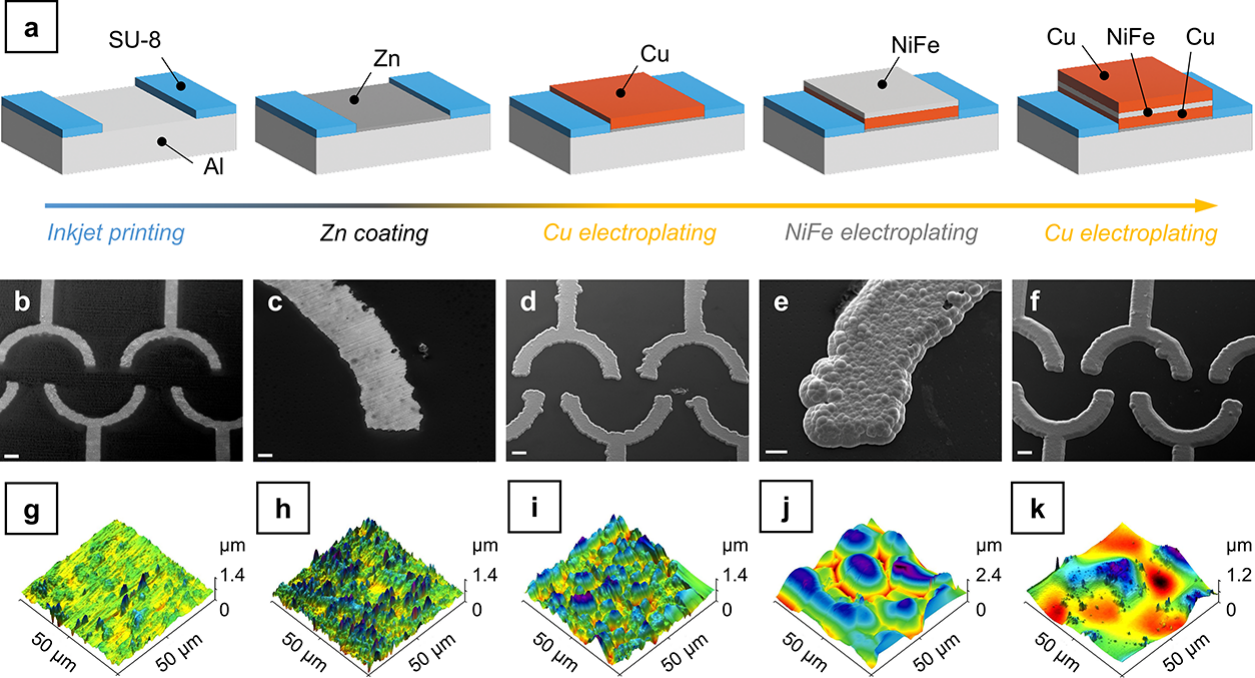
Schematic of manufacturing processes and morphology characterization results. (a) Manufacturing processes including inkjet printing for the template, Zn coating for the electrode, and electroplating of Cu and Ni–Fe. (b–f) SEM images in correspondence of each manufacturing step (scale bar: 200 μm for b, d and f; 40 μm for c and 60 μm for e). (g–k) AFM characterization results in correspondence of each manufacturing step.

Figure 2a visually depicts the production steps. Initially, SU-8 was patterned on the surface of the Al plate via inkjet deposition. The SU-8 pattern constituted the negative image of the devices and acted as a mask for metal electroforming. The bitmap employed to print the SU-8 layer, where each black pixel corresponded to a droplet ejected by the nozzle, is reported as Figure S1. At the end of the printing process, the resulting layer was reticulated and then employed as a mask for electroforming the devices. Al, however, cannot be directly employed as a substrate for copper electrodeposition in aqueous solutions. Indeed, this metal reacts in the most widely used Cu plating solutions based on acidic sulfates or alkaline pyrophosphates. This translates in lack of adhesion and bad quality of the final layer. Consequently, a double zincate process was employed to provide a Zn-rich coating able to support Cu deposition. Following the zincate step, Cu was deposited from a Cu pyrophosphate solution. Pyrophosphate-based solutions are characterized by a mildly alkaline pH, which is optimal for Cu deposition on the zincate coating. Indeed, the use of a highly acidic sulfate-based electrolyte would have resulted in possible damaging of the zincate layer itself and lack of adhesion. Following the first Cu layer, which had a structural function, NiFe was deposited to allow magnetic actuation. The composition of the electrolyte and the deposition parameters were selected to yield a nominal composition equal to 20 wt % Fe and 80 wt % Ni. These values correspond to the composition of the alloy commercially known as Permalloy. This material is characterized by a high magnetic permeability, by a low coercivity, and by a good saturation magnetization.^59,60^ It is therefore a good choice as a magnetic core for the propulsion of the microdevices under magnetic field gradients. The NiFe layer was covered with a second layer of Cu, deposited this time from a commercial sulfate-based electrolyte. Such electrolyte, in contrast with the pyrophosphate-based one, was highly additivated in order to provide a smooth Cu deposit. In fact, Cu deposition on NiFe could easily take place from the acidic sulfate bath and was employed to impart a low roughness finishing to the surface of the devices.

Following the last Cu electrodeposition step, SU-8 was stripped from the substrate, and the devices were subsequently removed from the Al surface by immersing it in a NaOH solution. Typically, it was not necessary to completely dissolve the substrate. The devices detached from the Al plate in a few hours, leaving a large part of the substrate not corroded. At the end of the process, the devices were easily collected via filtration.

SEM and AFM were carried out after each production step to understand the modifications progressively occurring on the devices. Figure 2b–f depicts the SEM morphology recorded in correspondence of each manufacturing phase. Figures 2b and S2 depict the morphology directly after SU-8 deposition. The shape of the negative mask, which was used as a template for electroforming, is clearly visible. The Al substrate, visible inside the SU-8 pattern, was characterized by a scratched morphology due to the polishing process employed to get a reproducible surface for subsequent electroforming. The printing lines left by the inkjet deposition process are clearly visible in Figure S2. Figure 2c depicts the morphology after the double zincate process. Since the zinc layer was relatively thin, surface morphology was not significantly altered by the zincate process. Figures 2d and S3 depict the morphology after the deposition of the first Cu layer. In this case, the morphology of the surface considerably changed, and the presence of a thick Cu layer is evident from Figure 2d. Since the pyrophosphate-based solution used to deposit the first Cu layer was not additivated, the surface roughness of the resulting material was relatively high (Figure S3). Figure 2e depicts the morphology after the deposition of the NiFe layer. The NiFe layer, which was characterized by a notably nodular morphology (Figure S4), was once again deposited from a nonadditivated solution. Consequently, the resulting layer was rough. Finally, Figures 2f and S5 depict the morphology after the deposition of the second Cu layer. Contrarily to the pyrophosphate solution, the sulfate-based solution employed to deposit this layer was additivated, resulting in a smooth Cu deposit that visibly decreased the roughness of the final device. Figure S6 depicts the OM appearance of an array of devices at the end of the second copper deposition step, prior to SU-8 stripping and Al substrate dissolution.

By looking at the SEM images (Figure 2b–f), it is evident that the dimensions of the devices changed after each step. Initially, the SU-8 mask was found to be smaller than the original design. As reported in Table 1, the width *w* of the negative images of the devices was measured in two different points (*w_1_* and *w_2_*), and it was equal to 180 ± 9.6 μm and 153.7 ± 8.6 μm in place of 200 μm. This effect is a direct consequence of the technique employed to pattern the mask. Inkjet printing, indeed, suffers from the so-called broadening effect.^52^ Each droplet deposited spreads on the substrate according to its wettability. Consequently, the droplets printed on the edges of the pattern tend to spread and decrease the inner width of the pattern itself. This effect can be counterbalanced by setting a gap between the edge of the pattern and the center of the droplets to compensate for spreading. In this case, however, the effect of pattern broadening was compensated by another phenomenon that took place during the electroforming step. Indeed, the dimensions of the devices increased by progressively depositing Cu, NiFe, and then again Cu. Each electrodeposited layer was deposited inside the SU-8 mask and partially grew beyond the border of the mask itself. The effect is clearly visible by measuring the width of the devices after each deposition step (Table 1). After the second Cu layer, the width of the devices was equal to 308 ± 4.4 μm, which is 154% of the expected dimension. The cross section reported in Figure S7 shows the final morphology of the three metallic layers. The part in contact with the Al substrate corresponded to the area originally present on the SU-8 mask. Two regions not in contact with the substrate are visible on the two sides. There, the metal grew on top of the mask.

**Table 1:** Dimensional Analysis for the Dimension *w* of the Devices after Each Production Step

	*w*_1_ (μm)	*w*_1_% vs nominal	*w*_2_ (μm)	*w*_2_% vs nominal
Nominal	200	100	200	100
SU-8 mask	180 ± 9.6	90	153.7 ± 8.6	76.8
1st Cu deposition	251.3 ± 8.5	125.7	224 ± 16.1	112
NiFe deposition	285 ± 4.6	142.5	251.3 ± 11.7	125.7
2nd Cu deposition	308 ± 4.4	154	290 ± 10.5	145

AFM was performed after each deposition step to evaluate the roughness and morphology variations occurring. Figure 2g–k shows the AFM morphology after each deposition step, respectively. Table 2, in turn, reports the values of roughness calculated from two different scan areas, namely, 50 × 50 μm and 20 × 20 μm.

**Table 2:** Surface Roughness Values after Each Production Step

	*R*_a_ (nm) on a 50 × 50 μm scan	*R*_a_ (nm) on a 20 × 20 μm scan
SU-8 layer	14.7 ± 1.4	15.3 ± 1.1
Al substrate	133.6 ± 12.2	53.8 ± 4.8
Al substrate + zincate	143.1 ± 11.3	96.7 ± 6.5
first Cu deposition	186.6 ± 17.3	190.8 ± 16.4
NiFe deposition	467.9 ± 55.9	318.6 ± 34.6
second Cu deposition	183 ± 11.0	69.6 ± 5.2

As already foreseen by looking at the SEM characterization previously discussed, roughness progressively increased moving from the uncoated Al substrate to the NiFe layer. Then, it considerably decreased after the deposition of the second Cu layer.

### 3.3 Device Characterization

**Figure 3:**
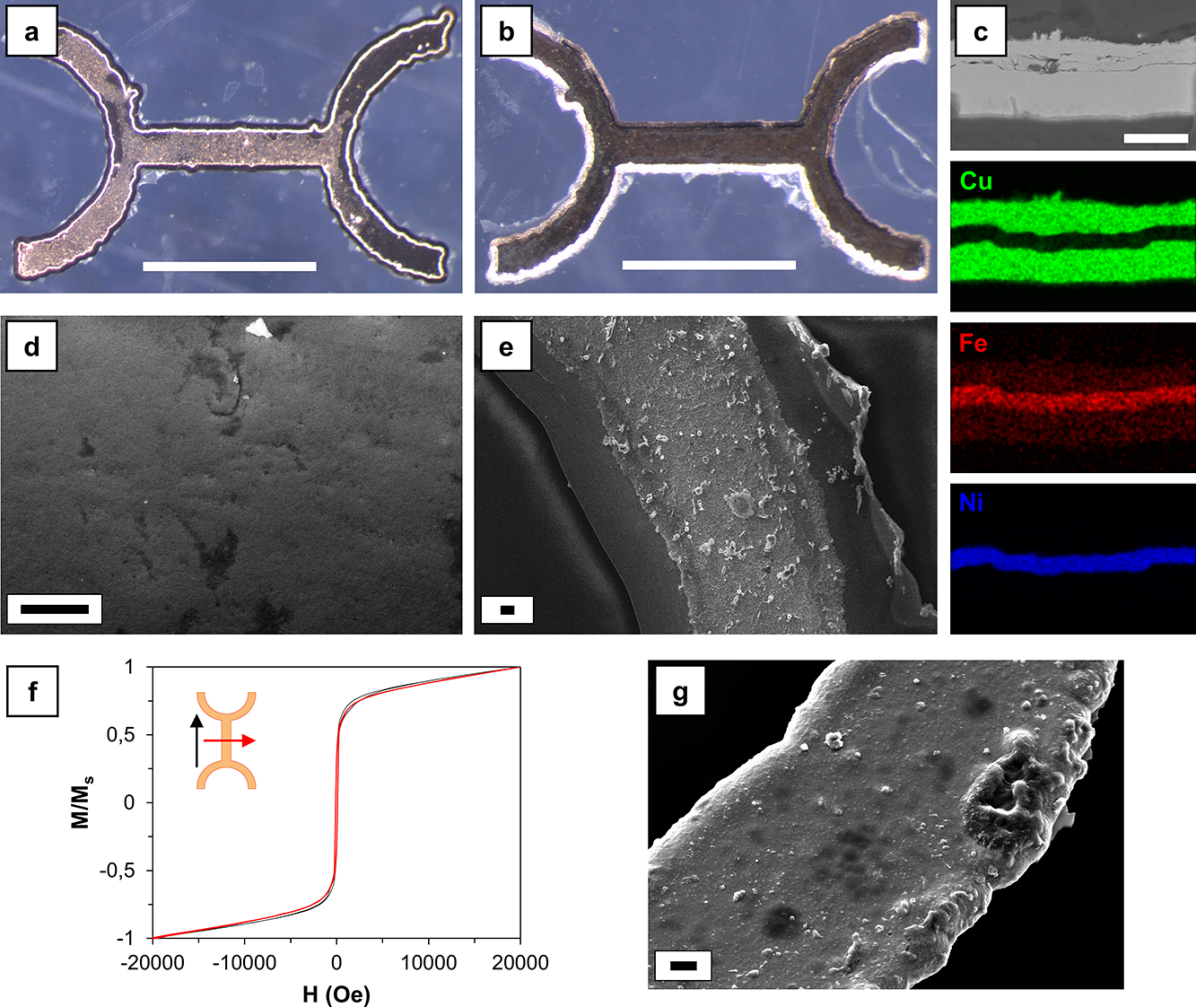
Characterization of the developed artificial water strider. (a) OM image of the top surface of the artificial water strider (scale bar: 1 mm). (b) OM image of the bottom surface of artificial water strider (scale bar: 1 mm). (c) Morphological SEM image and EDS elemental mapping for the cross section (scale bar: 20 μm). (d) SEM morphology of the top surface (scale bar: 10 μm). (e) SEM morphology of the bottom surface (scale bar: 10 μm). (f) Magnetization curve of the artificial water strider. (g) SEM image after coating AF1600 (scale bar: 10 μm).

At the end of the production process, the devices removed from the Al substrate were characterized to assess their morphological and magnetic properties. Figure 3a shows the morphology of a device observed at the OM, while Figure S8 depicts the equivalent SEM morphology. In analogy with what was recorded in the case of the width, all the dimensions of the devices slightly increased as a consequence of the electroforming process. Table S1 reports the experimental measures obtained from Figure 3a. Figure 3b shows the surface morphology of the bottom face of a device, which was in contact with the Al substrate. As expected, the morphology appears different.

Figure 3c depicts the cross-section and the corresponding elemental maps for Cu, Fe, and Ni of a single device removed from the Al substrate. Due to the broadening experienced by the metallic layers, their deposition area progressively increased during the deposition of the three layers. Consequently, being the current employed constant, current density progressively decreased, resulting in thicknesses lower than expected. In particular, the thickness of the first Cu layer was equal to 10.36 ± 0.86 μm. Since this was the first layer deposited and the initial area was the expected one, its thickness was in line with the design value. The situation changed with the NiFe layer and the second Cu layer. The first was characterized by a thickness equal to 5.17 ± 0.28 μm in place of 6 μm and the latter by a thickness equal to 7.86 ± 1.22 μm in place of 10 μm. This uneven internal distribution of the layers did not influence the mechanical properties of the device or its actuability. The magnetic force applied, indeed, mainly depends on the volume of magnetic material (eq 4) rather than on its distribution along the thickness of the device.

Figure 3d shows the SEM morphology of the top face of a device. The high smoothness of the last electrodeposited Cu layer is clearly visible. Figure 3e, on the contrary, depicts the lower face of a device, which was originally in contact with the Al substrate. Obviously, since it represents a negative image of the surface of the Al plate, its roughness is comparable. The two regions that grew on the SU-8 layer are clearly visible on the two sides of the bottom face. Regarding the composition of the surface, no Zn was detected on the bottom surface of the devices (Figure S9). This is not surprising, since Zn is not stable in highly alkaline solutions like the 2 M NaOH solution employed to dissolve the Al substrate (eq 5).
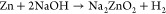
5

Consequently, the immersion in 2 M NaOH not only released the devices from the Al plate, but also removed the unwanted Zn from their lower face.

XRD was carried out (Figure S10) to characterize the NiFe alloy electrodeposited and to correlate its microstructure with the magnetic properties observed. The as-deposited alloy showed the characteristic peaks of a face-centered cubic (fcc) structure.^60^ The (111) and (200) reflections are clearly visible around 44° and 51°, respectively. The presence of a fcc structure constituted a first indication of the presence of a soft magnetic behavior, which was confirmed by the VSM analysis carried out subsequently. The devices, thanks to the presence of the permalloy layer, were ferromagnetic and characterized by the hysteresis loop reported in Figure 3f. It is worth noticing that the loop did not reach a plateau at high applied field, as expectable from NiFe.^60^ This can be reasonably explained considering the reduced mass of the devices, which yielded a weak signal that probably superimposed with a spurious paramagnetic component.^61^ The devices presented a coercivity (*H*_C_) equal to 87 Oe, which is indicative of a soft magnetic material. In addition, due to their geometrical anisotropy, the devices were characterized also by the presence of a preferential magnetization axis (also called easy axis). By looking at a magnification of the hysteresis loop (Figure S11), it can be immediately observed that the two hysteresis loops cannot be superimposed. On the contrary, the 0° direction (corresponding to the direction parallel to the axis of the device) presents slightly higher magnetization values. This is due to the distribution of the magnetic material in the microdevice and, in the presence of an external magnetic field, the device naturally orients itself along the 0° direction.

### 3.4 Surface Energy Control

As previously discussed, the buoyancy force acting on the device depends not only on the perimeter of the device itself but also on the surface energy γ between the solid material of the device and the fluid. Consequently, it is possible to maximize the force *F*_C_ by maximizing such surface energy via surface modification of the devices.^62^ Moreover, it is possible to adapt the device to the fluid by decreasing or increasing the surface energy. As-plated copper is characterized by a contact angle vs water of 93 ± 2.3° (Figure S12). This value is indicative of a relatively low surface energy, and in fact the devices easily floated on the surface of water. It is possible to tune the surface energy of the devices by applying suitable surface treatments. For example, the fluoropolymer AF1600 was applied by immersion on a device. The value of the contact angle obtained was 114 ± 3.1° (Figure S13). Figures 3g and S15 show the morphology of the device after the application of the AF1600 layer.

In the case of water, it is convenient to decrease the surface energy of the device. For other fluids, like many organic solvents, it is convenient to increase it. Consequently, the surface of the devices can be treated with suitable moieties or even simply subjected to annealing. It is well known, indeed, that the growth of an oxide layer in a controlled way allows considerably decreasing the surface energy of copper.^63^ In the present experimentation, a device was subjected to an annealing at 200 °C. The contact angle decreased to 25 ± 1.2° (Figure S14).

### 3.5 Actuation at the Water/Air Interface

To study their propulsion efficiency, as-plated devices were actuated at the water/air interface inside a circular water filled glass basin. Due to the presence of the glass walls, the shape of the water/air interface inside the basin was not flat. Indeed, water formed a meniscus in proximity of the walls. This phenomenon, which depends on the relatively low contact angle between water and glass, is detrimental for the movement of the devices. Dkhil et al. provided a deep insight of the phenomenon when they tried to move a magnetic particle toward the walls of a small water-filled basin.^64^ The meniscus, with its curvature, counterbalances the magnetic force acting on the device, which requires increasingly higher magnetic gradients to move. For this reason, the regions close to the walls of the basin are zones of high nonlinearity for what concerns the movement of the device. To avoid the effects induced by the presence of the meniscus, the region situated at a distance roughly equal to four times the height of the meniscus itself^65^ was not used for actuation tests.

**Figure 4:**
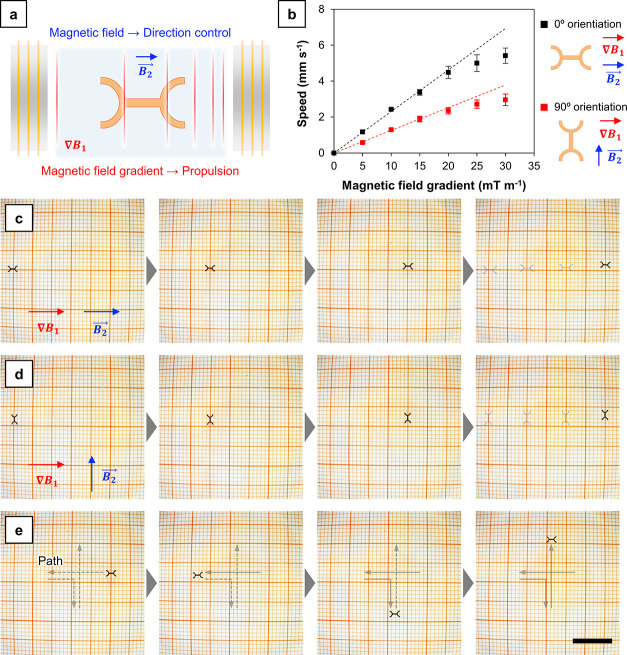
Navigation of the artificial water strider by using magnetic field. (a) Schematic diagram showing the actuation principle of an artificial water strider. (b) Change of linear speed as a function of applied magnetic field gradient. (c) Linear actuation of a single device with 0° orientation and (d) 90° orientation. (e) Navigation following the cross-shaped path (scale bar: 10 mm).

Consequently, the devices were actuated keeping them distant from the walls. The devices were found to move easily on the surface of the water and relatively weak gradients of few mT m^–1^ were enough to reach significant speeds. Obviously, the devices moved in the same direction of the gradient, but it was found that the orientation of the device could be controlled independently from its direction of motion. This was possible because the Octomag setup, which was used to actuate the devices, was able to independently apply a gradient and a constant magnetic field along two different directions. Figure 4a exemplifies the situation: the gradient ∇*B*_1_ is applied along a certain direction, resulting in the force *F*_m_, but the device presents its magnetic easy axis oriented along the direction determined by the angle θ thanks to the magnetic field *B*_2_. The easy axis rotates and follows the applied field because the latter is able to generate a torque *T*_m_ on the device^2^ (eq 6).
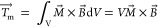
6

It is evident that the torque applied on the device does not depend on the gradient of the field applied but only on its orientation. Consequently, the device can be oriented independently from the direction of its motion by applying a constant field along an arbitrary direction. For example, Figure 4c and Supporting Video 1 describe the motion of a device when the gradient of *B*_1_ (equal to 20 mT m^–1^) has the same orientation of the constant field *B*_2_ (equal to 1 mT). In this case, the axis of the device, which corresponds to the easy axis for magnetization, is aligned with the direction of the movement. In Figure 4d and Supporting Video 2, on the contrary, the θ angle between the gradient of *B*_1_ and *B*_2_ was equal to 90°, and the axis of the device was consequently turned of 90° with respect to the direction of the motion. Also in this case, the gradient was set to 20 mT m^–1^ and the constant field to 1 mT.

The speed of the devices could be controlled varying the intensity of the gradient applied. For example, Supporting Video 3 shows the actuation of a device under the influence of a 5 mT m^–1^ gradient, and its speed is visibly lower than that of the device actuated at 20 mT m^–1^ (Supporting Video 1). The speed of the devices with respect to the gradient applied was evaluated, and the results obtained are plotted in Figure 4b. To calculate the speed, a single device was actuated linearly at constant applied gradient, and its position was tracked. In general, the devices were able to reach considerably high speeds even when relatively low gradients were applied. Under a 30 mT m^–1^ field, for example, the devices reached a speed up to 5.41 mm s^–1^, which corresponded to more than two body length per second. In addition to this first consideration, two other interesting peculiarities can be highlighted in Figure 4b. First, the orientation of the device has a great influence on the speed. Second, the speed does not increase linearly, but it tends to deviate from linearity for gradients higher than 20 mT m^–1^. To understand the behavior observed, it is useful to qualitatively evaluate the influence of the drag exerted by the water on the speed of the device. To do this, the well-known drag equation can be considered (eq 7). Theoretically, it is valid for a fully immersed object and floating objects require more complex treatises.^66,67^ However, it is conceptually valid to qualitatively explain the influence of fluid drag on the water striders described in the present work.
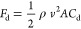
7

The drag force, *F*_d_, depends on the density of the fluid (ρ), the speed of the object (*v*), the cross-sectional area of the object (*A*), and the drag coefficient (*C*_d_). In general, the drag force opposes to the magnetic force acting on the device and limits the final speed. The nonlinearity of the speed can be explained by the fact that the drag force does not increase linearly with the speed. Indeed, it is characterized by a square dependence from the speed, whereas the magnetic speed linearly increases with the gradient applied (eq 4). When the speed is comparatively high, therefore, the contribution of the drag force is higher, leading to a visible nonlinearity. Regarding the different speed observed between the two orientations (0° and 90°), the effect can be easily rationalized by looking again at eq 7. The drag force depends on the cross-sectional area of the device *A*, which is higher when the device is oriented at 90° with respect to the direction of its motion. Consequently, also the force that opposes to the magnetic actuation is higher.

Obviously, the direction of the motion can be controlled by varying the direction along which the gradient is applied. Figure 4e and Supporting Video 4 depict a multidirectional actuation obtained by applying the gradient first along the −*x* direction, then along the *+x* direction, and immediately turning it toward the −*y* direction. Finally, the direction was reverted, and the device moved along the *y* direction. The gradient was varied between 0 and 10 mT m^–1^, and the direction was set with a 1 mT constant field. The controllability of the devices was in general high, with the possibility of performing arbitrary patterns on the surface of the water.

### 3.6 Oil Droplet Collection at the Water/Air Interface

The devices described in the present work can be employed to manipulate small objects floating at the interface between two fluids,^68^ or even droplets of a third fluid floating at the interface between two other fluids. The last scenario is of notable interest for a variety of applications: oil collection at the water–air interface, controlled microreactions inside droplets, spreading of fluids layers at interfaces, and manipulation of self-assembled monolayers. Two of these possible applications were demonstrated to give an applicative perspective to the devices developed in the present work. The first was oil collection at the water/air interface.

**Figure 5:**
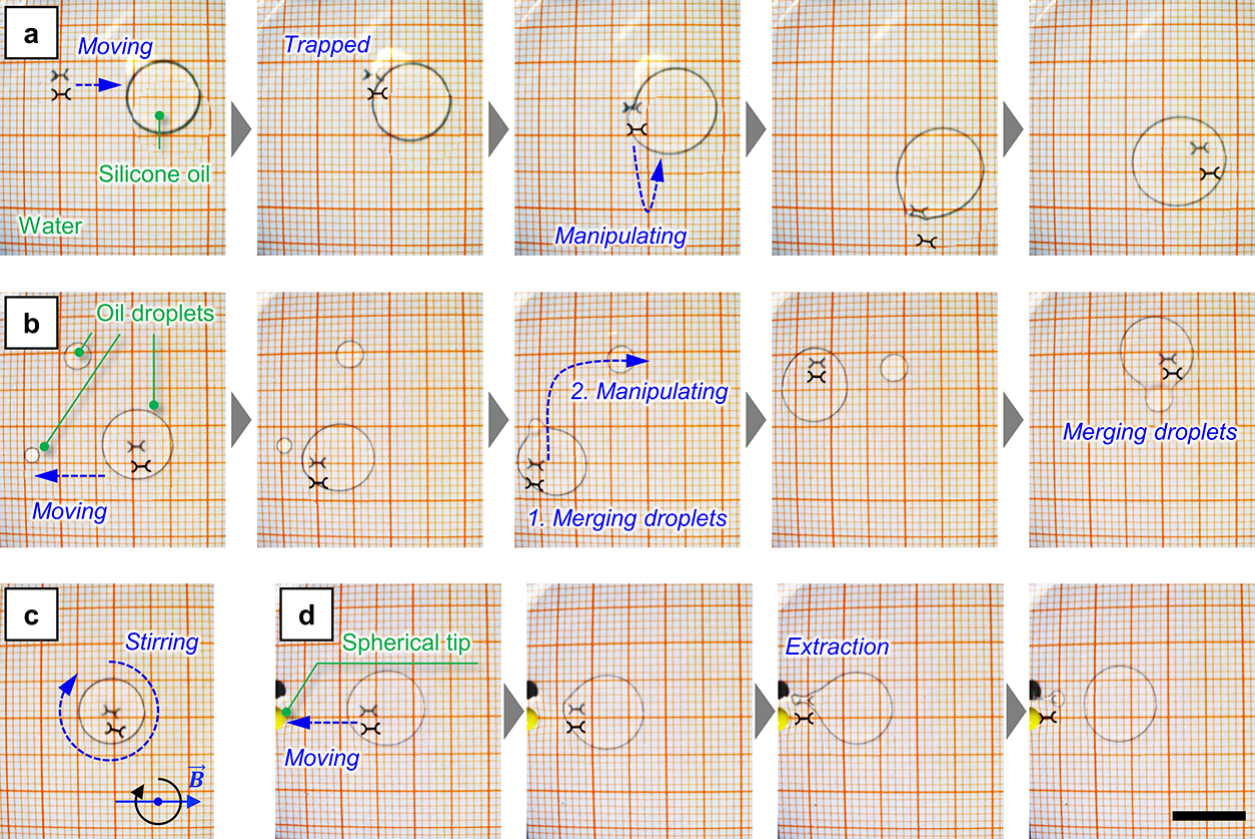
Demonstration of oil droplet manipulation. (a) Sequential images showing the inglobation of an artificial water strider into a droplet of siliconic oil. (b) Sequential images showing the collection of multiple oil droplets. (c) Frame showing the rotation of a device inside an oil droplet. (d) Sequential images showing the meniscus favored separation of an oil droplet from a device (scale bar: 10 mm).

The scope of the experiment was to controllably move and collect droplets of silicone oil floating on the water/air interface. In this case, the silicone oil was characterized by a density lower than water (0.95 g cm^–3^), and it floated on the interface between water and air in the form of droplets. Consequently, it was possible to collect it by placing a AF1600 modified device on the water and guiding it toward the droplets. The interaction between a single droplet of oil and the device is visible in Figure 5a and Supporting Video 5. The device touched the surface of the droplet and, due to its low surface energy, started interacting with the silicone oil. It was slowly inglobated by the droplet itself and trapped inside. Once inglobated, the device was able to drag the droplet and controllably move it (Supporting Video 6). By moving the device, and consequently the droplet in which it was inglobated, isolated droplets of oil could be collected by approaching them. As visible in Figure 5b and Supporting Video 7, two isolated droplets were collected by touching and merging them with the large droplet guided by the device.

In addition, if the external constant field rotates, it is possible to continuously generate a torque on the device and apply a rotation (Figure 5c and Supporting Video 8). In this way, it is possible to stir the fluid inside the droplet. Finally, it is also possible to separate the oil from the device by exploiting the presence of a meniscus. In particular, a polymeric spherical tip was immersed in correspondence of the edge of the basin and the device was guided toward it (Figure 5d and Supporting Video 9). The tip created a meniscus, which was climbed by the device thanks to the magnetic force. During this motion, due to gravity, the device left behind the droplet of oil.

### 3.7 Chemical Microreactions in Dynamically Guided Droplets

The second application was the management of controlled microreactions inside droplets. A large number of studies on this topic are available in the literature, demonstrating the appeal of the approach. Indeed, reactions carried out inside droplets allow optimal material consumption and a highly controllable reaction environment. In the case of the devices described in the present paper, it is possible to carry out reaction in an aqueous environment by carefully selecting the relative viscosities of the fluids involved. By using a high-density silicone oil, it is possible to have the water-based fluids floating on an organic phase instead of the opposite situation described in the previous example. By doing this, it is possible to place on the surface of silicone oil multiple droplets of water-based solutions, which can be remotely manipulated using the devices. This is the ideal situation to carry out microreactions inside droplets.

**Figure 6:**
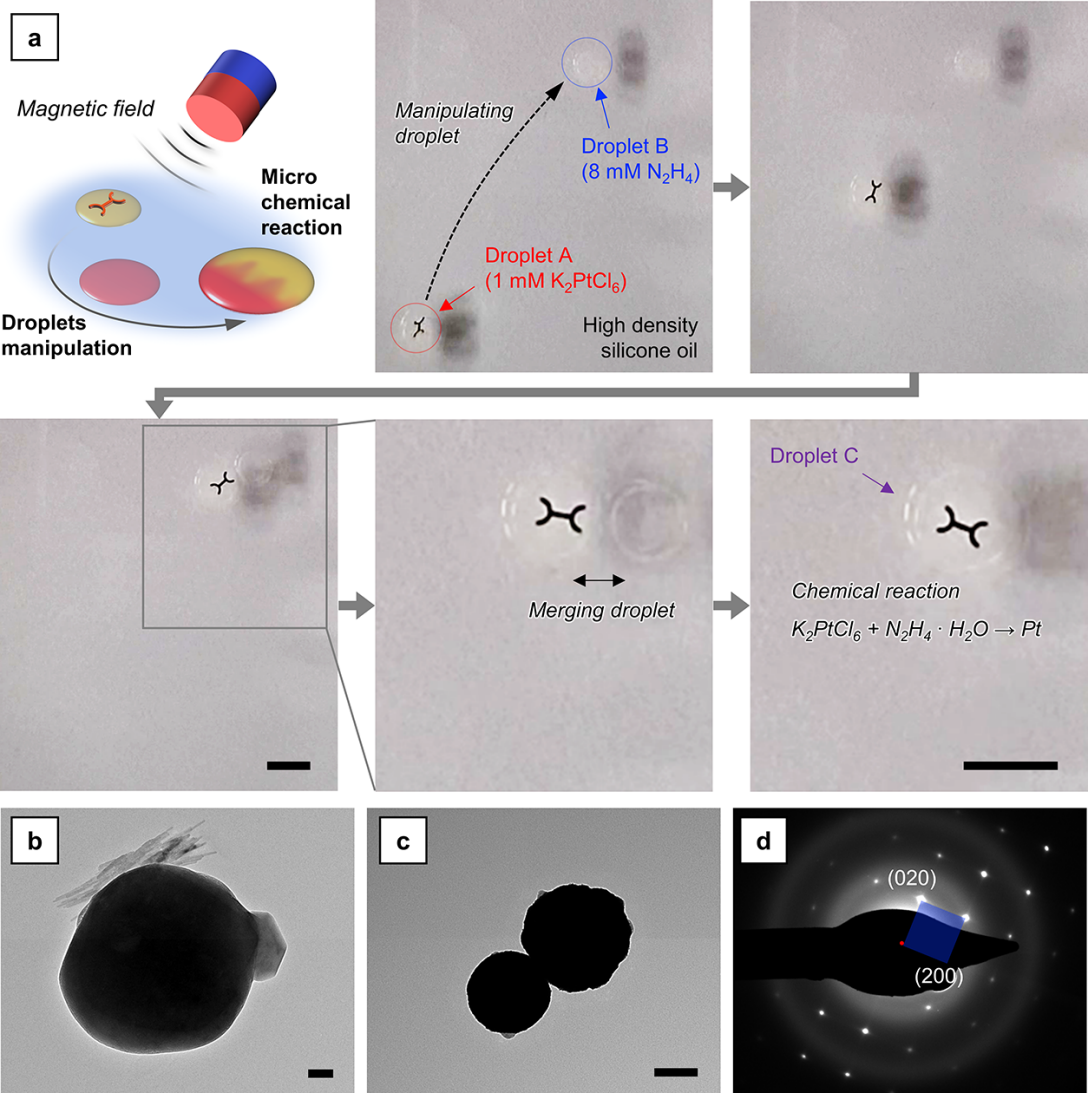
Demonstration of droplet-based chemical microsynthesis. (a) Sequential images showing that a magnetic-field-guided microrobot performs the microreaction experiment for the synthesis of Pt NPs (scale bar: 5 mm). (b) TEM image of the particles synthesized from the macroreaction (scale bar: 100 nm). (c) TEM image of the particles synthesized from the microreaction (scale bar: 100 nm). (d) SAED pattern of the particles synthesized from the microreaction.

As a case study, the synthesis of platinum NPs was performed (Figure 6a and Supporting Video 10). A droplet of solution A (containing Pt^4+^) was dispensed on the surface of a silicone oil-filled basin. Then, a droplet of solution B (containing hydrazine) was dispensed. Both solutions were prepared using a 50 vol % ethanol mixture with water as solvent. In this way, the density of the solutions was considerably lower than that of pure water (0.928 g mL^–1^ in place of 0.998 g mL^–1^ at 20 °C), resulting in the formation of well-shaped droplets on the surface of the silicone oil. An annealed device was placed on the surface of the silicone oil and guided toward the droplet of solution A. As soon as it touched the surface of the droplet, the device was inglobated and the droplet could be easily moved. Droplet A was moved and placed in contact with droplet B. After a few seconds, the two merged and the reaction took place forming droplet C. Droplet C was moved and then collected using a micropipette. For comparison, the same reaction carried out using the droplets was done using macroscopic volumes of the two solutions A and B.

The results of the two approaches were analyzed using TEM. As expectable from the reaction between diluted K_2_PtCl_6_ and hydrazine solutions,^69,70^ the morphology of the NPs was globular. Figure 6b reports the result obtained from the macroreaction, while Figure 6c depicts the result obtained from the microreaction. The shape of the NPs was similar, but their mean diameter was slightly different: 350–400 nm for the particles produced from the macroreaction and 200–250 nm for the particles produced from the microreaction. This difference can be ascribed to the different fluid dynamic conditions in the two systems. In both cases, the Pt NPs obtained were characterized by a monocrystalline structure, as visible in the SAED pattern reported in Figure 6d. Two crystallographic orientations of the fcc lattice of Pt are clearly visible in Figure 6d, (020) and (200).

## 4 Conclusions

In the present work, artificial water striders were successfully designed, manufactured, and magnetically actuated at the interface between representative fluids. Moreover, their potential applicability has been experimentally validated presenting two possible uses. The inkjet-assisted electroforming process employed for their manufacturing proved capable of reproducing patterns with significant precision, allowing the production of devices containing features down to the few hundreds of micrometers as the dimensional range. At the end of the manufacturing cycle, the surface of the water striders was modified to adapt its wettability to the fluid required by the specific application. In the case of high-surface tension fluids, like water, the wettability of the devices was decreased by applying a fluoropolymer. For low-surface tension fluids, like silicone oil, the contact angle of the surface was manipulated by growing a controlled oxide layer. Following these two approaches, the relative wettability between the surface and the liquid was constantly maximized, allowing optimal buoyancy. The presence of an electrodeposited NiFe layer between the two structural layers that constitute the body of the device allowed direct wireless manipulation by means of external magnetic fields. Indeed, magnetic manipulation proved able to precisely control the speed and the position of the devices. From the applicative point of view, two conceptual applications were demonstrated: oil collection at the water/air interface and microreaction management in water droplets at the oil/air interface. The fluoropolymer-functionalized devices proved highly efficient in gathering oil droplets scattered on the surface of a body of water. Multiple droplets were collected by contacting them with the device and then guided in the desired location. Oxidized devices, on the other hand, proved optimal to carry out small-scale reactions inside droplets of water floating on the surface of silicone oil. These were merged in a controlled way, allowing the reaction between a platinum salt and a reducing agent to yield platinum NPs. Besides these two applications, artificial water striders may potentially find use for the manipulation of floating objects or for the spreading of monolayers at the interface between two fluids. In addition, since the devices present a certain load capacity in terms of weight, it is also possible to load further functional layers or even electronic circuits on their surface. In this way, environmental or monitoring applications could be theoretically implemented.

## Supporting Information Available

Design and dimensions of the devices; bitmap images used to print the SU-8 layer; SEM of the SU-8 layer; SEM of the first copper layer; SEM of the NiFe layer; SEM of the second copper layer; OM of an array of microdevices; SEM section of a device; SEM of the top face of a device; EDS of the bottom face of a device; XRD of the NiFe alloy; details of the hysteresis cycle for a single device; contact angle measurement for water on as-plated Cu; contact angle measurement for water on AF1600-coated Cu; contact angle measurement for water on annealed Cu; and SEM of an AF1600-coated device (PDF)
Video depicting the motion of a device when the gradient of *B*_1_ (equal to 20 mT m^–1^) has the same orientation of the constant field *B*_2_ (equal to 1 mT) (MP4)
Video showing the θ angle between the gradient of *B*_1_ and *B*_2_ equal to 90° (MP4)
Video depicting the actuation of a device under the influence of a 5 mT m^–1^ gradient (MP4)
Video showing multidirectional actuation obtained by applying the gradient first along the −*x* direction, then along the *+x* direction, and immediately turning it toward the −*y* direction (MP4)
Video showing visible interaction between a single droplet of oil and the device (MP4)
Video depicting the device dragging the droplet and controllably moving it after inglobation (MP4)
Video depicting two isolated droplets collected by touching and merging them with the large droplet guided by the device (MP4)
Video showing continuous generation of a torque on the device and applying a rotation (MP4)
Video depicting a polymeric spherical tip immersed in correspondence of the edge of the basin with the device guided toward it (MP4)
Video showing synthesis of platinum NPs (MP4)
