# Supplementary material for: Inkjet-Assisted Electroformation of Magnetically Guidable Water Striders for Interfacial Microfluidic Manipulation
Source: ACS Appl Mater Interfaces. 2022 Dec 13;15(1):2396–408. doi: 10.1021/acsami.2c17792 (PMC9837820; doi:10.1021/acsami.2c17792)
Supplement: Supplementary file 1 — am2c17792_si_001.pdf [file am2c17792_si_001.pdf]

Supporting Information for:

Inkjet assisted electroforming of magnetically guidable water striders for interfacial microfluidic manipulation

*Roberto Bernasconi[a] *, Davide Carniani[a], Min-Soo Kim[b], Salvador Pané[b], Luca Magagnin[a]*

[a] Dipartimento di Chimica, Materiali e Ingegneria Chimica "Giulio Natta", Politecnico di Milano, via Mancinelli 7, 20131, Milano (Italy)

[b] Multi-Scale Robotics Lab, Institute of Robotics and Intelligent Systems, ETH Zurich, Tannenstrasse 3, CH-8092, Zürich (Switzerland)

* Corresponding author: roberto.bernasconi@polimi.it

Table S1 reports the nominal dimensions of the devices and compares them with the experimental ones measured at the end of the manufacturing process.

Table S1. Nominal vs. experimental dimensions of the devices.

**Dimension**	**Nominal value (μm)**	**Experimental value (μm)**
**L**	2500	2512 ± 8
**l**	1000	1046 ± 6
**D**	1500	1532 ± 10
**d**	1100	1061 ± 9
**w**	200	279 ± 4

Figure S1 is the bitmap employed to print the SU-8 layer on the Al substrate during the first production step. Each black pixel corresponds to a droplet jetted by the Dimatix printer.

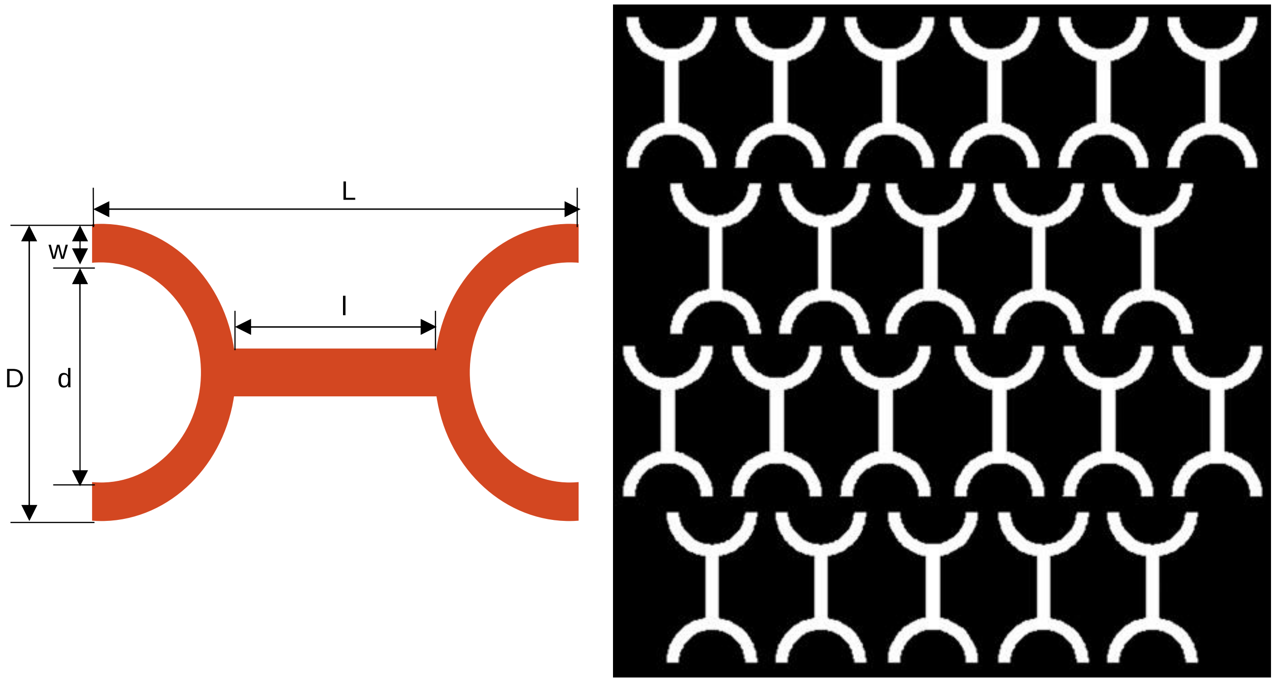

Figure S1. Design of the artificial water strider and its bitmap image employed to print the SU-8 layer

Figure S2 shows the SEM of the SU-8 layer. Figure S3 reports the SEM after the deposition of the first Cu layer from a pyrophosphate electrolyte.

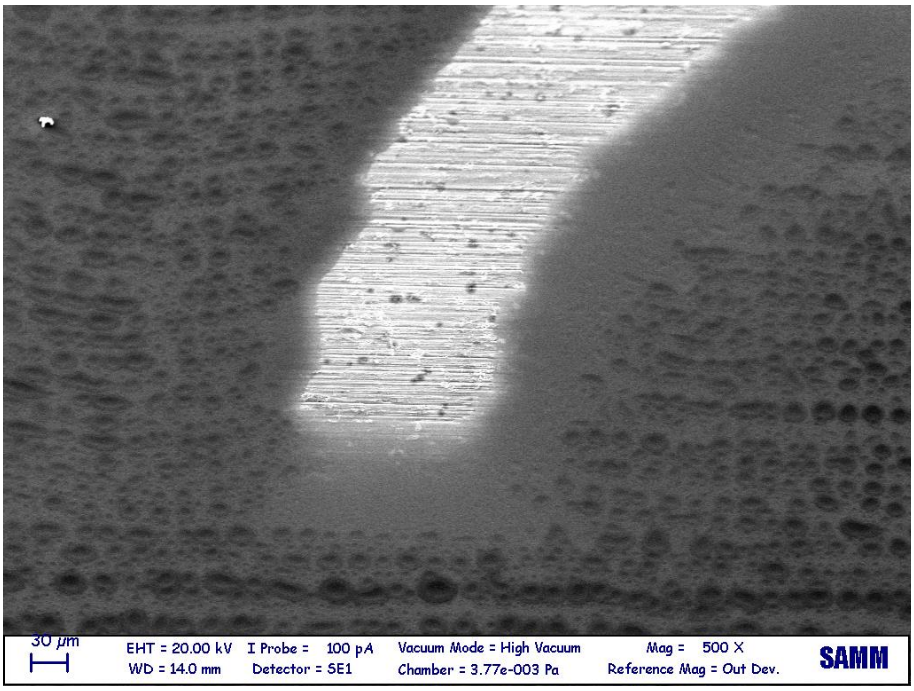

Figure S2. SEM of the SU-8 layer

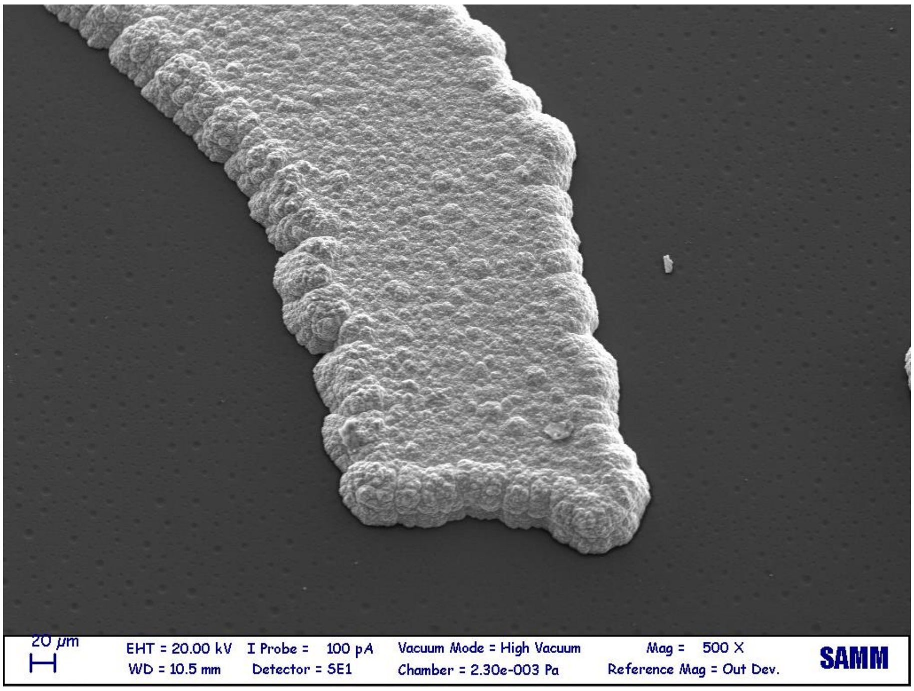

Figure S3. SEM of the first copper layer

Figure S4 shows the SEM of the NiFe layer. Figure S5 reports the SEM after the deposition of the second Cu layer from a sulfate electrolyte.

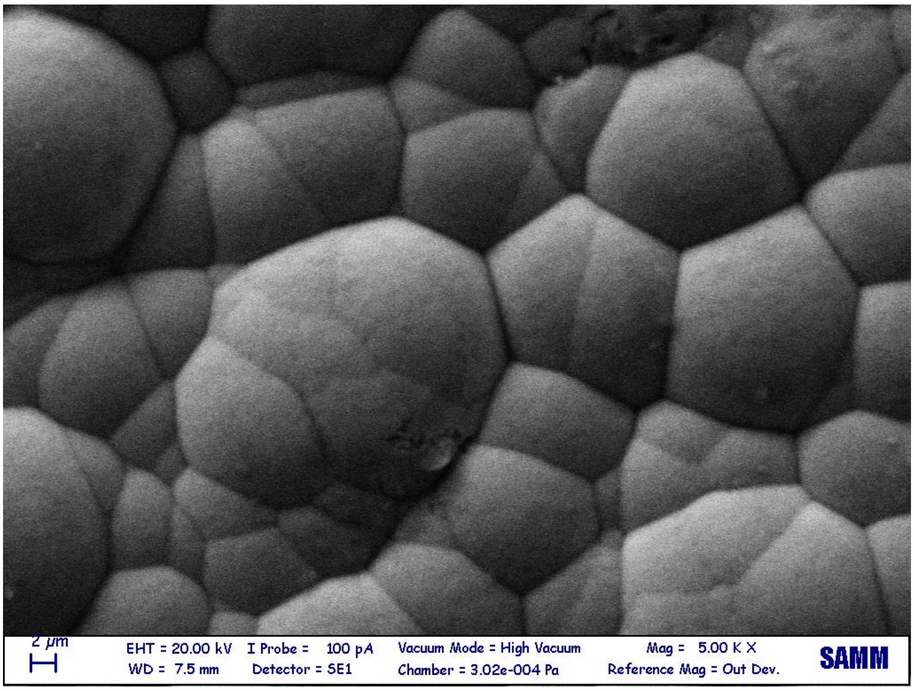

Figure S4. SEM morphology of the NiFe layer

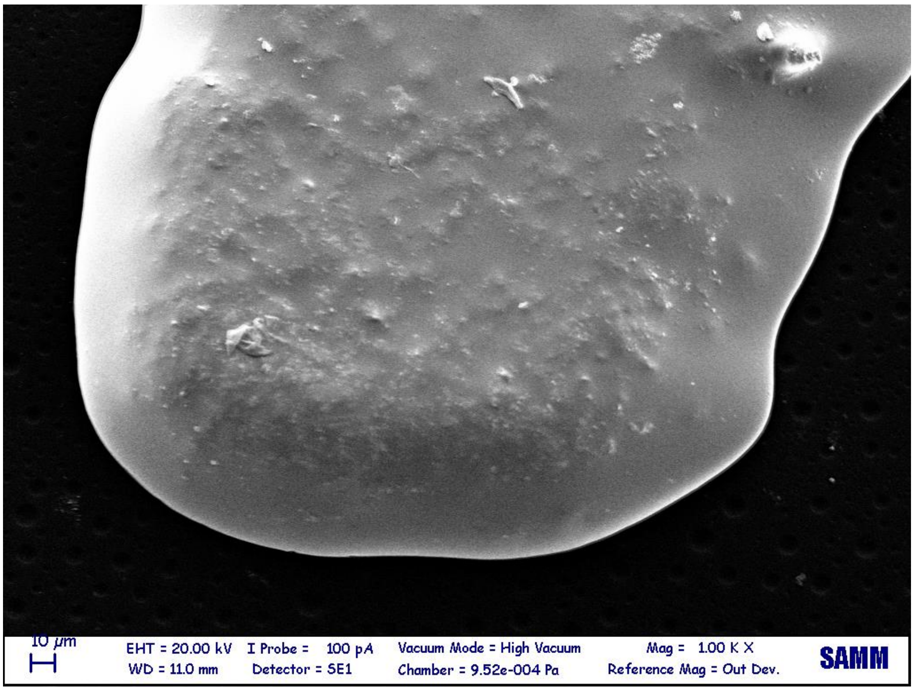

Figure S5. SEM morphology of the second copper layer

Figure S6 reports the OM of an array of devices immediately after the deposition of the second Cu layer. Figure S7 depicts the SEM of the section of a device.

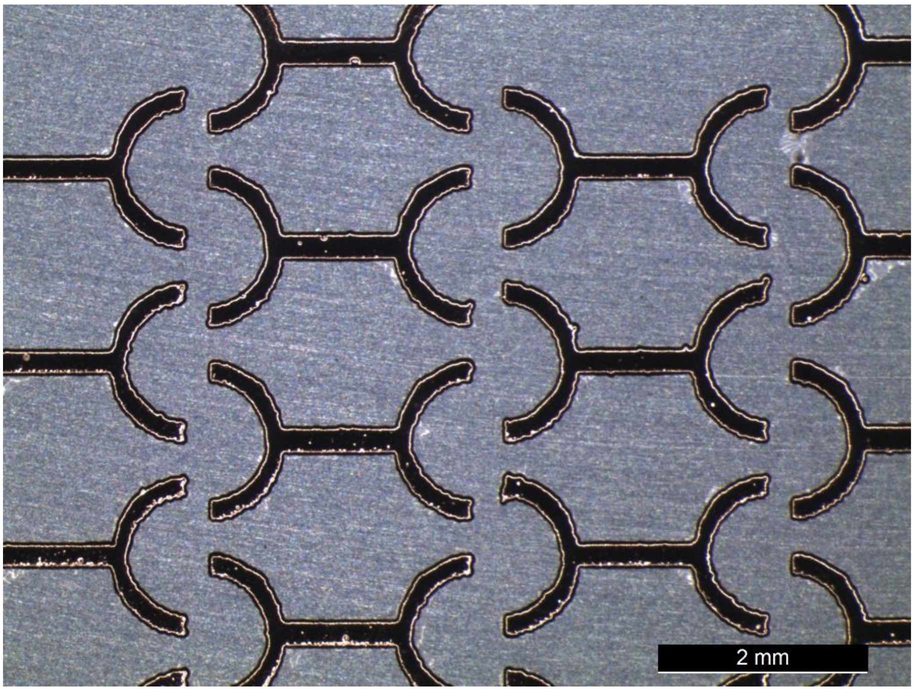

Figure S6. OM of an array of microdevices still in contact with the Al substrate

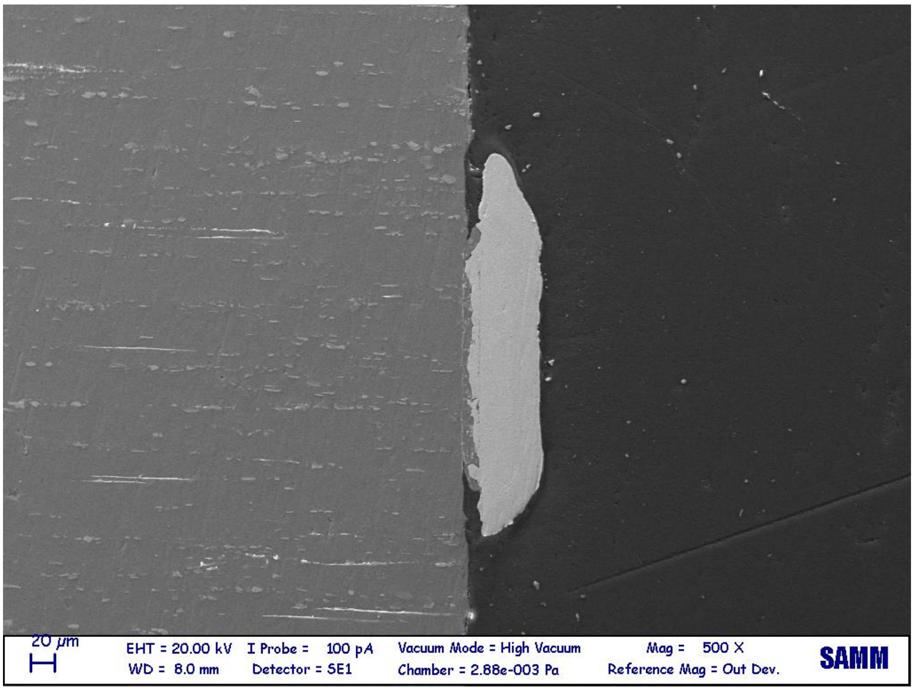

Figure S7. SEM section of a device

Figure S8 reports the SEM of the top surface of a detached device. Figure S9 describes the EDS analysis of the bottom face of a detached device.

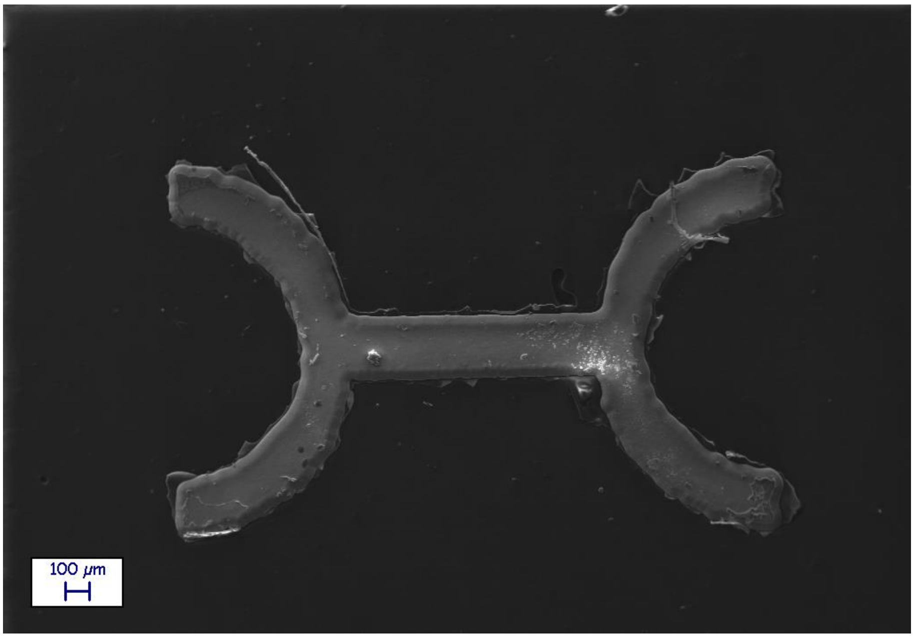

Figure S8. SEM of the top face of a device

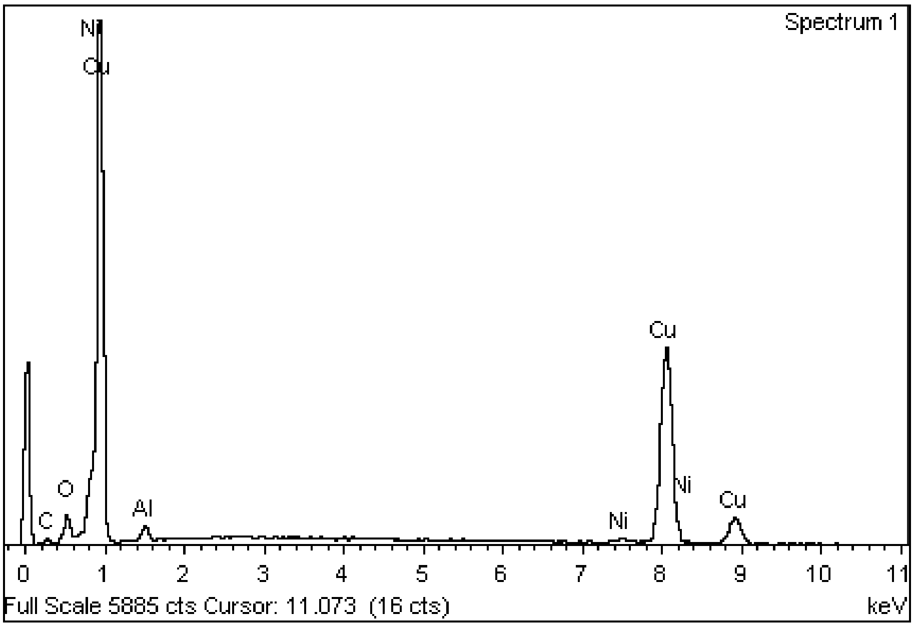

Figure S9. EDS of the bottom face of a device

Figure S10 reports the XRD of the NiFe alloy. Figure S11 is a magnification of the hysteresis cycle presented in Figure 3f of the main text.

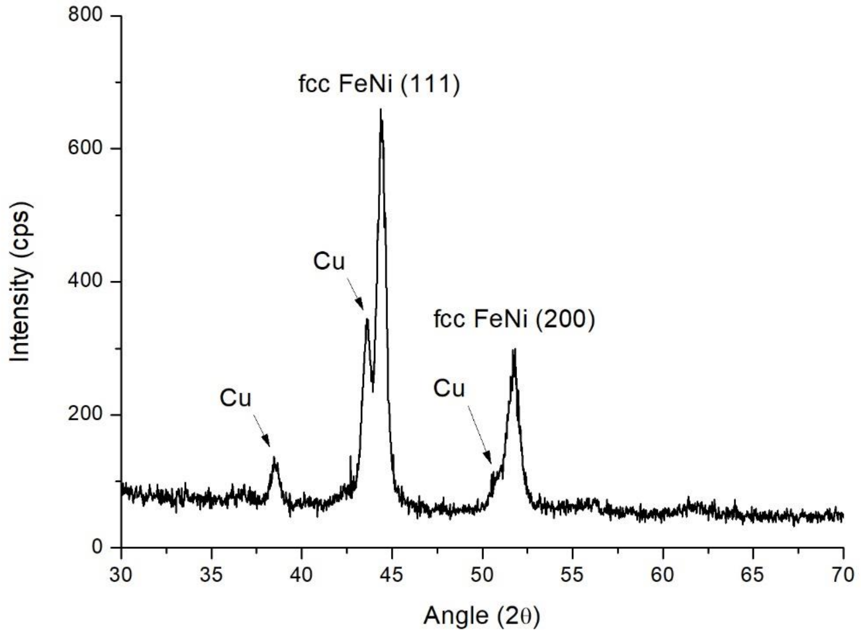

Figure S10. XRD of the NiFe alloy

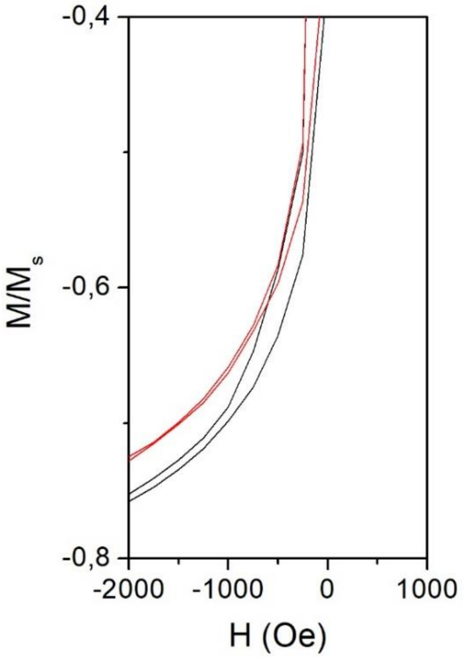

Figure S11. Magnification of the hysteresis cycle for a single device (red correspond to the direction parallel to the axis of the device; black corresponds to the direction perpendicular to the same axis)

Figure S12-S14 show the contact angle (CA) for as-plated Cu, AF1600 treated Cu and annealed Cu, respectively.

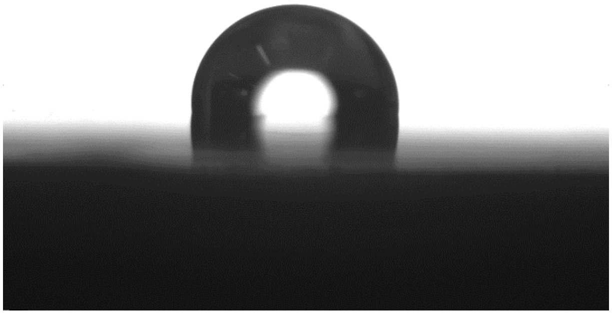

Figure S12. CA for water on as-plated Cu

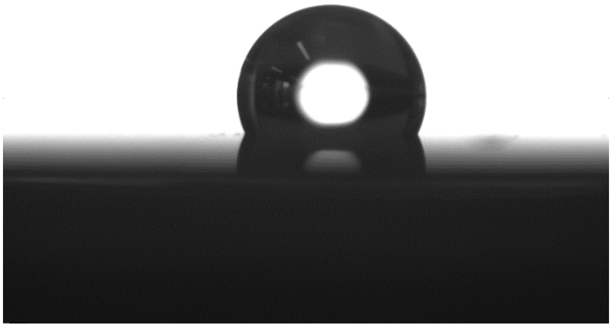

Figure S13. CA for water on AF1600 coated Cu

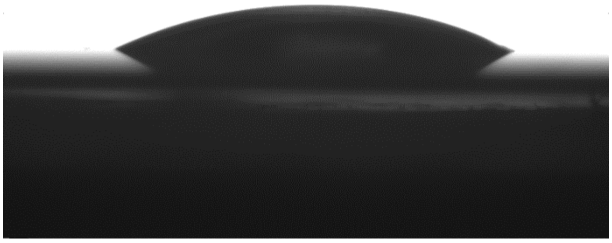

Figure S14. CA for water on annealed Cu

Figure S15 shows the SEM characterization of a device coated with a layer of AF1600.

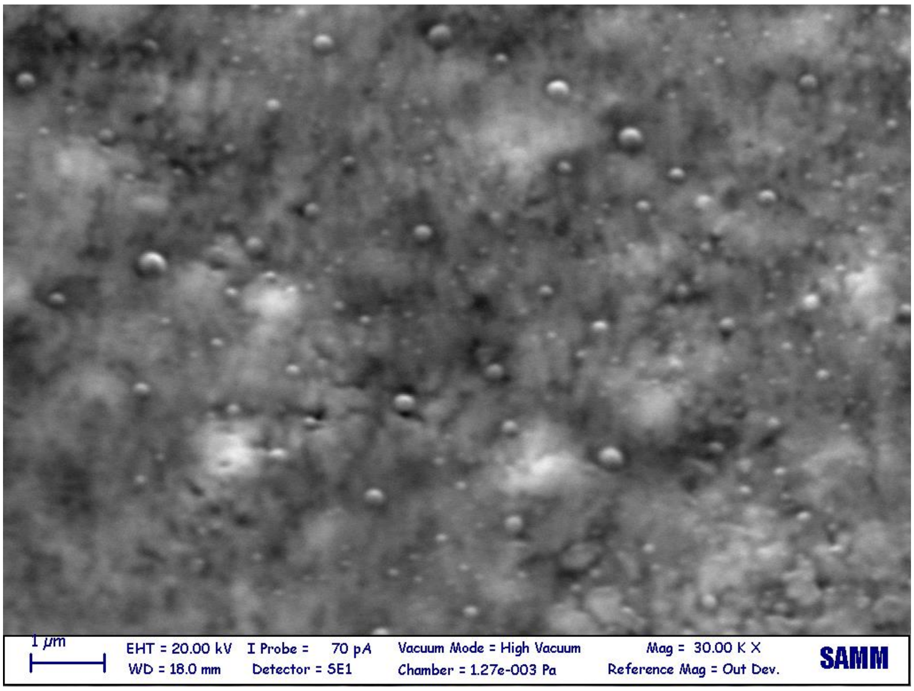

Figure S15. SEM of a AF1600 coated device
